# The giant E3 ligase HUWE1 is linked to tumorigenesis, spermatogenesis, intellectual disability, and inflammatory diseases

**DOI:** 10.3389/fcimb.2022.905906

**Published:** 2022-07-22

**Authors:** Lu Qi, Xiaoqing Xu, Xiaopeng Qi

**Affiliations:** ^1^ Department of Orthopedics, The Second Affiliated Hospital of Shandong University of Traditional Chinese Medicine, Jinan, China; ^2^ Department of Oncology, Affiliated Hospital of Shandong University of Traditional Chinese Medicine, Jinan, China; ^3^ Key Laboratory for Experimental Teratology of the Ministry of Education, Department of Clinical Laboratory/Qilu Hospital, Advanced Medical Research Institute, Cheeloo College of Medicine, Shandong University, Jinan, China

**Keywords:** HUWE1, ubiquitination, tumorigenesis, DNA damage response, inflammasome

## Abstract

E3 ubiquitin ligases determine the substrate specificity and catalyze the ubiquitination of lysine residues. HUWE1 is a catalytic HECT domain-containing giant E3 ligase that contains a substrate-binding ring structure, and mediates the ubiquitination of more than 40 diverse substrates. HUWE1 serves as a central node in cellular stress responses, cell growth and death, signal transduction, etc. The expanding atlas of HUWE1 substrates presents a major challenge for the potential therapeutic application of HUWE1 in a particular disease. In addition, HUWE1 has been demonstrated to play contradictory roles in certain aspects of tumor progression in either an oncogenic or a tumor-suppressive manner. We recently defined novel roles of HUWE1 in promoting the activation of multiple inflammasomes. Inflammasome activation-mediated immune responses might lead to multifunctional effects on tumor therapy, inflammation, and autoimmune diseases. In this review, we summarize the known substrates and pleiotropic functions of HUWE1 in different types of cells and models, including its involvement in development, cancer, neuronal disorder and infectious disease. We also discuss the advances in cryo-EM-structural analysis for a functional-mechanistic understanding of HUWE1 in modulating the multitudinous diverse substrates, and introduce the possibility of revisiting the comprehensive roles of HUWE1 in multiple aspects within one microenvironment, which will shed light on the potential therapeutic application of targeting giant E3 ligases like HUWE1.

## Introduction

HUWE1 is a giant E3 ligase that functions as a central node in cellular stress responses, cell growth and apoptosis. HUWE1 is involved in signaling pathways, transcriptional regulation, neuronal differentiation, spermatogenesis, tumorigenesis, DNA damage responses, etc. Furthermore, the *Huwe1* gene is located on the X chromosome and associated with X chromosome-linked intellectual disability. Recently, we defined HUWE1 as a master regulator of inflammasome activation and recognized its novel roles in the fields of inflammatory responses and infectious diseases. In this manuscript, we review the roles of HUWE1 in multiple aspects, discuss the controversial finding that HUWE1 can be considered either an oncogene or tumor suppressor, and present the challenges in therapeutic strategies targeting giant E3 ligases such as HUWE1.

## Ubiquitination and E3 ligases

Ubiquitination is an evolutionarily conserved modification in which ubiquitin moieties are added to lysine residues of substrates in all eukaryotic organisms. The process of ubiquitination is mediated by three key enzymes: E1 ubiquitin-activating enzymes, E2 ubiquitin-conjugating enzymes and E3 ubiquitin ligases (Scheffner et al., 1995). The E3 ubiquitin ligase determines the substrate specificity and catalyzes the formation of isopeptide bonds between ubiquitin moieties and lysine residues of substrates. More than 600 E3 ubiquitin ligases are encoded by the human genome and are classified into three major categories: really interesting new gene (RING)-type, homologous with E6-associated protein C-terminus (HECT)-type, and RING-between-RING (RBR)-type (Berndsen and Wolberger, 2014). RING-type E3 ligases mediate ubiquitin transfer from E2 enzymes directly to the substrate. In contrast, HECT-type and RBR-type E3 ligases first transfer the ubiquitin moiety from the E2 enzyme to a catalytic cysteine on the E3 enzyme and then to the substrate (Morreale and Walden, 2016). Ubiquitination is one of the most important posttranslational modifications (PTMs) that mediates the degradation, intracellular localization and interaction of proteins. Ubiquitination plays major roles in regulating many cellular processes, such as cell division, cell differentiation, cell death, and signal transduction, which depend on the type of polyubiquitin chain (M1, K6, K11, K27, K29, K33, K48, or K63) (Tracz and Bialek, 2021). K48- and K11-linked polyubiquitin chains have been attributed to proteasome-mediated proteolysis of the substrate (Ravid and Hochstrasser, 2008). K63- and M1-linked polyubiquitin chains are involved in the processes of signaling and innate immune responses (Chen, 2012). K6-, K27-, K29-, and K33-linked polyubiquitin chains have rarely been investigated and mostly thought to modulate signaling, protein trafficking, DNA damage and innate immune responses (Akutsu et al., 2016).

## HUWE1 is an X chromosome-linked giant E3 ligase

**Figure 1:**
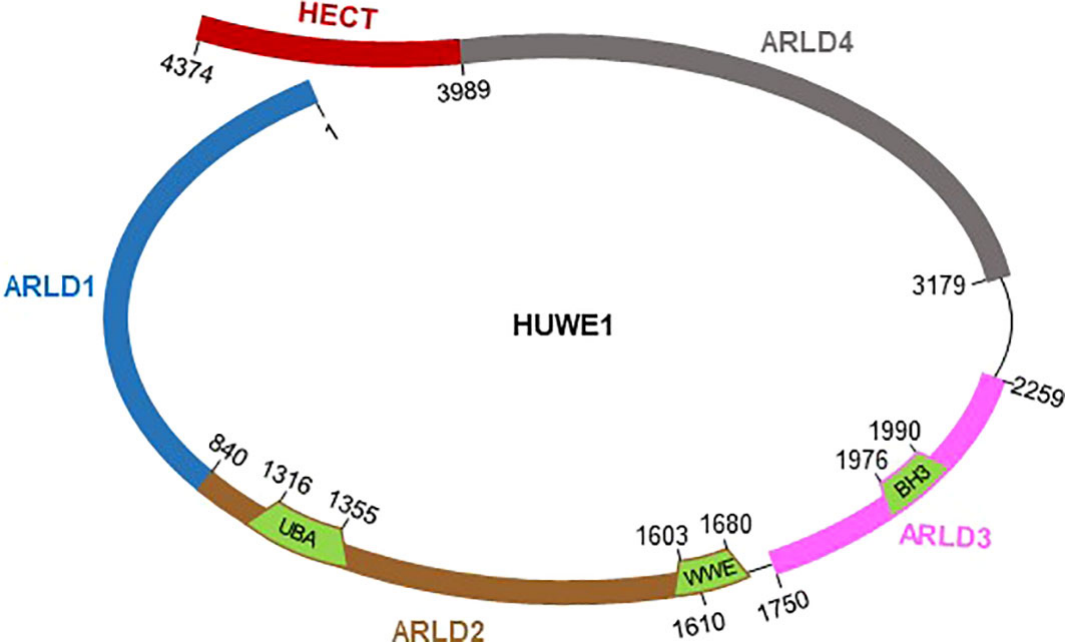
Schematic representation of full-length HUWE1 with functional domains and indicated boundaries. HUWE1 harbors a ring-shaped architecture composed of four armadillo repeat-like domains (ARLD1-4), and a flexible C-terminal HECT catalytic domain.

HECT, UBA and WWE domain-containing protein 1 (HUWE1), also called ARF-BP1, MULE, HECTH9, LASU1, and UREB1, is a large E3 ligase with 4374 aa in humans and 4378 aa in mice (Warr et al., 2005; Giles and Grill, 2020). HUWE1 was initially detected in a screen of large cDNA clones from the brain (Nagase et al., 1997), and full-length cDNA was identified and studied by five independent groups in 2005 (Adhikary et al., 2005; Chen et al., 2005; Liu et al., 2005; Yoon et al., 2005; Zhong et al., 2005). The HUWE1 protein is evolutionarily conserved across the animal kingdom from *C. elegans* to humans (Giles and Grill, 2020), and the identity of the HUWE1 protein sequence between humans and mice is more than 90% (Zhong et al., 2005). HUWE1 in humans contains four N-terminal armadillo repeat-like domains (ARLD1-4) and a C-terminal HECT ubiquitin ligase domain (**Figure 1**). A ubiquitin-associated domain, UBA; a WWE domain involved in ubiquitin-dependent proteolysis; and a BH3 domain responsible for protein-protein interactions are identified within ARLD1-4 (Zhong et al., 2005; Hunkeler et al., 2021). Recently, remarkable progress has been made in understanding the control of HECT ligase activity and substrate specificity of the HUWE1 protein. Due to its large size, the HUWE1 protein has been identified to mediate the ubiquitination of a multitude of diverse substrates through its giant substrate-binding ring structure, and this ring structure is highly dynamic. The ligase activity of the flexible C-terminal HECT domain is actually determined by its conformational change through dimerization of the C-terminal HECT domain or disruption of the dimer interface (Sander et al., 2017; Grabarczyk et al., 2021; Hunkeler et al., 2021). Furthermore, *Huwe1* gene is located on the X chromosome and associated with X chromosome-linked intellectual disability. Recently, we defined HUWE1 as a master regulator of inflammasome activation. Next, we review the roles of HUWE1 in multiple aspects and discuss the challenges in therapeutic strategies targeting the substrate-selective activity of giant E3 ligases such as HUWE1.

## HUWE1 regulates the DNA damage response and apoptosis

Through a two-hybrid screen for proteins interacting with Cdc6, Hall et al. identified the HECT-family ubiquitin E3 ligase HUWE1, which directly interacted with Cdc6 and ubiquitinated Cdc6 for degradation in response to DNA damage. Of note, this was the first study that used the official gene name HUWE1 for the HECT, UBA and WWE domain containing 1 gene (Hall et al., 2007). In addition, the authors demonstrated conserved HUWE1-mediated degradation of Cdc6 in budding yeast. The HUWE1 ortholog TOM1 in yeast was also found to regulate Cdc6 degradation. This study indicated that HUWE1 acts as a tumor suppressor by mediating Cdc6 degradation and driving cell cycle arrest and apoptosis during the DNA damage response. Previously, Zhong et al. had also reported that HUWE1 (Mule, Mcl-1 ubiquitin ligase E3) mediated the proteasomal degradation of Mcl-1 and was required for DNA damage-induced apoptosis; combining these results, HUWE1 was considered to be a proapoptotic protein (Zhong et al., 2005). HUWE1 activity should be tightly regulated to shut down apoptosis induced by DNA damage, thus promoting the DNA damage repair process and cell survival. Indeed, the E3 ligase CUL4B was observed to interact with HUWE1 and ubiquitinate it for proteasomal degradation in response to DNA damage (Yi et al., 2015).

HUWE1 was reported to target and regulate the activity of chromosome-associated proteins such as histone proteins, H2AX, and histone modification proteins during the DNA damage response. HUWE1 mediates the ubiquitination and degradation of H2AX in normal cells in a quiescent state to maintain rapid H2AX turnover. H2AX is transiently stabilized and forms γH2AX foci upon DSB formation *via* the ATM kinase and SIRT6/SNF2H blocking HUWE1 activity (Atsumi et al., 2015). HUWE1 was also reported to interact with the important DNA replication factor PCNA and mediate H2AX monoubiquitination and phosphorylation to promote DNA damage repair signaling at stalled forks. Thus, HUWE1-deficient cells exhibited increased DNA breakage and genomic instability (Choe et al., 2016). Furthermore, HUWE1 was reported to prime ubiquitination of histone H1, which is an essential and important signaling intermediate in the UV-induced DNA damage response (Mandemaker et al., 2017). The histone acetyltransferase Myst2 is critical for the maintenance of pluripotency and self-renewal in embryonic stem cells, and the stability of Myst2 needs to be decreased for proper cell differentiation. HUWE1 was recently reported to be a novel ubiquitin ligase of Myst2 during embryonic stem cell development, and HUWE1-mediated ubiquitination of Myst2 was found to be blocked by Brpf3 overexpression (Cho et al., 2020). HUWE1 was demonstrated to modulate the DNA damage repair pathway by directly regulating DNA polymerase activity. DNA polymerase β is an important base excision repair (BER) enzyme for the removal of DNA lesions during the DNA damage response. ARF-BP1/Mule was identified to interact with polymerase β and catalyze its ubiquitination for degradation. Thus, ARF-deficient cells exhibit decreased DNA repair capacity and an increased probability of tumor development (Parsons et al., 2009). In addition, Mule was found to mediate proteasomal degradation of the DNA polymerase Pol 𝜆, which counteracted the phosphorylation of Pol 𝜆 involved in chromatin binding and oxidative DNA damage repair (Markkanen et al., 2012). Overall, these findings indicate that HUWE1-mediated proteasomal degradation of chromatin-binding proteins contributed significantly to the regulation of gene transcription and genome integrity.

The Chk1 kinase is one of the major cell cycle regulators that functions downstream of DNA damage repair signaling. HUWE1 can regulate the stability of Chk1, and HUWE1 knockdown was found to result in increased endogenous DNA damage and prolong the half-life of Chk1 (Cassidy et al., 2020). In addition, DNA damage-inducible transcript 4 (DDIT4) was identified to interact with HUWE1 and be ubiquitinated for degradation through quantitative diGly proteomics in control and HUWE1 knockdown cells (Thompson et al., 2014). In an ischemia-reperfusion model, proper expression of growth arrest and DNA damage-inducible protein 45 beta (Gadd45b) was found to be essential for recovery. Inhibition of HUWE1 by shRNA increased the expression of Gadd45b in neurons treated with oxygen-glucose deprivation (OGD) and reperfusion, and HUWE1 was proposed to interact with Gadd45b for its ubiquitination and degradation (He et al., 2015). In addition to mediating ubiquitination, HUWE1 was also reported to mediate DNA-PKcs neddylation, which contributed to its phosphorylation and repair activity in the DNA damage response (Guo et al., 2020b). In multiple myeloma (MM) models, increased HUWE1 expression was found to be associated with MM disease progression. HUWE1 contributed to tumor growth by promoting endogenous DNA damage repair, and targeting HUWE1 might be a potential therapeutic approach for MM (Kunz et al., 2020). Recently, through a genome-wide CRISP/Cas9 screening system, HUWE1 was identified among the top hits that correlated with cisplatin sensitivity. Deficiency of HUWE1 can result in an increased DNA damage response, dysfunctional apoptotic cell death, etc. Thus, HUWE1 has critical roles in modulating cisplatin sensitivity (Wenmaekers et al., 2021).

## HUWE1 regulates cell proliferation and tumor development

The expression levels of HUWE1 are frequently higher in certain cancers, such as lung cancer and leukemia, than in the corresponding normal tissues. However, HUWE1 expression is reduced in glioblastoma and sarcoma (Confalonieri et al., 2009; Kao et al., 2018; Su et al., 2019). In fact, HUWE1 can act as either an oncogene or tumor suppressor depending on the type of cancer (Kao et al., 2018), and the association between HUWE1 expression and overall survival is not clear for most cancers (Su et al., 2019).

Ubiquitination is a dynamic modulation crucial for tumor-suppressing or tumor-promoting processes in various types of cancers through modulation of different substrates. Substrate specificity is achieved through E3 ligases, and a wide survey of E3 ligase dysregulation in cancer revealed HUWE1 as a significantly correlated prognostic factor. The well-known substrates of HUWE1 in tumor development are the Myc and p53 proteins. HUWE1 (named HectH9) was initially identified as a Myc-interacting protein by screening the interaction partners of Miz, a repressive Myc-interacting protein. HectH9 was reported to catalyze K63-linked polyubiquitination of Myc and function as an oncogene to mediate the transcriptional activation of a subset of Myc target genes to promote tumor cell proliferation (Adhikary et al., 2005). However, in prostate cancer cell lines, HUWE1 overexpression was found to inhibit cell proliferation, cell migration and explant growth, and c-Myc was downregulated in human prostate cancer (CaP) cells with high HUWE1 expression (Qu et al., 2018), suggesting that HUWE1 functioned as a tumor suppressor by negatively regulating c-Myc activity. In colorectal cancer, hUREB1 (HUWE1) was initially determined to inhibit the activity of p53 and promote tumor development. Furthermore, an inverse correlation between HUWE1 and p53 expression in colorectal carcinoma progression was identified, suggesting that HUWE1 mediated the ubiquitination and proteasomal degradation of p53, although the direct interaction between HUWE1 and p53 was not defined in that study (Yoon et al., 2005). In a study to identify ARF-mediated tumor suppressors, Chen et al. observed that the ARF-associated protein ARF-BP1 (HUWE1) directly bound and ubiquitinated p53, which reduced the stabilization of p53 (Chen et al., 2005). In B cells, ARF-BP1 (HUWE1) was found to mediate K48-linked polyubiquitination of p53 for its degradation and K63-linked polyubiquitination of Myc for its activation. Overexpression of Myc reversed the defects-induced by ARF-BP1 deficiency. The dynamic balance between p53 and Myc mediated by ARF-BP1 was essential for normal B cell maturation and lineage commitment (Qi et al., 2013). HUWE1 expression was downregulated in thyroid cancer samples compared with control samples. HUWE1 knockdown promoted cell proliferation, migration and invasion. In thyroid cancer, HUWE1 was found to function as a tumor suppressor by increasing p53 protein stabilization (Ma et al., 2016). The function of HUWE1 in tumor growth or inhibition rarely depends on the cell type and is mediated through different mechanisms. The expression of HUWE1 in multiple myeloma (MM) cells was increased compared with that in normal cells, and was significantly correlated with the expression of Myc. HUWE1 was reported to positively regulate the activity of Myc, possibly through metabolic regulation of intracellular glutamine homeostasis (Crawford et al., 2020), and Myc degradation might result from the lower level of glutamine in HUWE1-deficient cells. In addition, HUWE1 inhibition by the inhibitors BI8622 and BI8626 reduced the proliferation of MM cells, and HUWE1 inhibition promoted the antitumor effects of conventional MM therapies.

Mcl-1 is an anti-apoptotic Bcl-2 family member protein, and its degradation and elimination are essential and required for DNA damage-induced apoptosis. Zhong et al. identified MULE (HUWE1) as a BH3-containing E3 ubiquitin ligase. They further demonstrated that MULE directly bound to Mcl-1 at five lysine residues for its ubiquitination, and promoted its proteasomal degradation (Zhong et al., 2005). The interaction between HUWE1 and Mcl-1 was also defined through position weight matrix (PWM) analysis in a study to identify new members of the BH3-containing Bcl-2 family in HeLa cells. In this study, LASU1 (HUWE1) was found to bind to Mcl-1 and mediate its proteasomal degradation. The protein level of Mcl-1 was increased in the context of LASU1 depletion by siRNA knockdown (Warr et al., 2005). In addition, deficiency of Mule in B cells at steady state resulted in increased p53 accumulation, and Mule maintained p53 at a basal level to support B cell development and homeostasis. However, in response to genotoxic stress, the Mule protein interacted with phospho-ATM, and the protein levels of phospho-ATM, phospho-p53, and Brca1 in B cells were reduced in the absence of Mule, leading to an apoptosis resistance phenotype, suggesting that Mule maintained B cell homeostasis under both steady-state and stress conditions (Hao et al., 2012). In non-small-cell lung cancer development, HUWE1 was also found to target p53 for proteasomal degradation and control the development of cancer. The authors revealed that high expression of HUWE1 was associated with poor survival in lung cancer patients (Yang et al., 2018). Obesity and IL-6 increase the incidence of hepatocellular carcinoma development through Mcl-1 stabilization and apoptosis suppression. In obesity, the expression of HUWE1 was found to be suppressed, leading to stabilization of Mcl-1 independent of IL-6Ra signaling, which contributed to liver carcinogenesis (Gruber et al., 2013). In addition to modifying Mcl-1, MULE/HUWE1/ARF-BP1 was reported to abolish the interaction between c-Myc and Miz1. HUWE1 mediated K48-linked polyubiquitination of both c-Myc and Miz1 for degradation, which resulted in increased p21 and p15 levels to support cell cycle arrest and senescence. In addition, skin tumorigenesis in HUWE1-deficient mice was reversed by c-Myc deficiency, and the increased keratinocyte proliferation in *Huwe1*-deficient mice was reversed by Miz1 deficiency (Inoue et al., 2013). This study demonstrated that both c-Myc and Miz1 are direct targets of HUWE1 and that HUWE1 functions as a tumor suppressor. Moreover, Miz1 was found to be essential for topoisomerase II binding protein 1 (TopBP1) binding to chromatin and activating Atr-dependent signal transduction. Myc inhibited the interaction of TopBP1 with Miz1 and promoted HUWE1-mediated proteasomal degradation of TopBP1. Thus, HUWE1-mediated TopBP1 stability was determined to be important for the interaction between Miz1 and Myc for Atr checkpoint activity (Herold et al., 2008). In an intestinal tumor model with loss of the tumor suppressor gene *Apc*, HUWE1 deficiency resulted in increased Myc protein expression, accumulation of DNA damage and elevated Mcl-1 protein expression, which drove accelerated intestinal tumorigenesis. This study identified HUWE1 as functioning as a *bona fide* tumor suppressor by inhibiting tumor initiation (Myant et al., 2017).

In epigenetic regulation, Mule (HUWE1) was reported to target histone deacetylase 2 (HDAC2) for ubiquitination and proteasomal degradation. In HUWE1-deficient cells, increased HDAC2 impaired p53 acetylation and activity, leading to compromised DNA damage-induced apoptosis (Zhang et al., 2011). This study revealed HUWE1 as functioning as a critical tumor suppressor by modulating the cellular apoptotic response to HDAC inhibition and DNA damage. The clinical relevance of HUWE1 expression to ovarian cancer tumorigenesis was analyzed by using immunohistochemical staining with an anti-HUWE1 antibody and RNA analysis. A high expression level of HUWE1 was observed in the majority of tumors and was correlated with high mortality, suggesting that a high level of HUWE1 is a negative predictive and prognostic factor in ovarian cancer treated with chemotherapy (Podgorska et al., 2015). Mechanistically, Yang et al. demonstrated that HUWE1 promoted ovarian tumor growth through downregulation of H1.3 and upregulation of H19 expression by directly interacting with H1.3 and mediating its degradation, which sustained ovarian epithelial cell transformation and tumor growth without affecting cell survival and apoptosis (Yang et al., 2017). In addition, in B-cell lymphoma, ARF-BP1 (HUWE1) was identified to be a component of the multiprotein complex composed of HUWE1, ARF, p53, MYC and the multifunctional nuclear factor CTCF. HUWE1 mediated K48-linked polyubiquitination of CTCF for proteasomal degradation, which significantly regulated both the Myc proliferative and p53 apoptotic pathways (Qi et al., 2012).

Wnt signaling plays an important role in the development and homeostasis. The Dishevelled (Dvl) protein is a master regulator of upstream events in the Wnt pathway, and Dvl multimerization is thought to be critical for regulating Wnt signaling activity. HUWE1 and its homolog in *C. elegans*, EEL-1, were demonstrated to mediate Dvl ubiquitination and suppress Dvl multimerization and Wnt signaling (Page et al., 2007; De Groot et al., 2014). This study further showed the conserved role of HUWE1 in mediating the ubiquitination of proteins in the Wnt signaling pathway and provided evidence that K63-linked polyubiquitination suppresses multimerization, a finding that was different from the results of other studies showing that K63-linked ubiquitination mostly mediated the activation of signaling. MyoD is a master regulator of muscle development that activates a broad array of muscle-specific genes. HUWE1 was identified to interact with MyoD and contribute to its ubiquitination and degradation (Noy et al., 2012). BRCA1 is a tumor suppressor and plays important roles in the DNA damage response by modulating checkpoint activation and DNA repair. The protein level of BRCA1 is tightly regulated through a number of mechanisms. HUWE1 was reported to interact with BRCA1 and mediate its ubiquitination and degradation (Wang et al., 2014). In addition to affecting transcription factors and nuclear proteins, HUWE1 also modulates the activity of cell membrane proteins and receptors to influence carcinoma development. HUWE1 mediated the ubiquitination and degradation of the RAC activator TIAM1, which abolished cell-cell adhesion and promoted lung cancer cell invasion and dissemination (Vaughan et al., 2015). In tubulointerstitial fibrosis, opposite changes in HUWE1 and EGFR expression were found in the kidneys of unilateral ureteral obstruction (UUO) mice. Indeed, HUWE1 was observed to directly interact with EGFR, promote its ubiquitination and degradation, and prevent renal tubulointerstitial fibrosis (Zhu et al., 2020).

Mitochondrial metabolism is essential for modulating many processes linked to oncogenesis and has attracted considerable attention for the development of novel anticancer therapies (Porporato et al., 2018). The expression of HectH9 (HUWE1) has been associated with disease progression in prostate cancer. Recently, HectH9 was reported to mediate K63-linked polyubiquitination of HK2, leading to mitochondrial localization of HK2 and a tendency toward glycolysis. In addition, glucose deprivation caused apoptosis in the absence of HectH9, which inhibited tumor metabolism and cancer stem cell expansion (Lee et al., 2019). This was the first report to show that HectH9 regulates tumor cell pathogenesis by targeting metabolic pathways. Moreover, HUWE1 was found to regulate HIF-1α-dependent tumorigenesis. TiPARP is an ADP ribosyltransferase that forms nuclear condensates or nuclear bodies, which recruit both the oncogenic transcription factor HIF-1α and the E3 ubiquitin ligase HUWE1. HUWE1 was found to mediate the ubiquitination and degradation of HIF-1α to suppress tumorigenesis (Zhang et al., 2020). Under hypoxic conditions, HectH9 was found to mediate the K63-linked polyubiquitination and activation of HAUSP (USP7), and HIF-1α was subsequently deubiquitinated and stabilized to further promote EMT and metastasis. Indeed, coexpression of HectH9 and HIF-1α was identified as a prognostic marker in lung cancer cases (Wu et al., 2016). The balance of mitochondrial fission and fusion is a highly dynamic process for the maintenance of mitochondrial function. Mitochondrial outer membrane permeabilization (MOMP) and mitochondrial fragmentation are associated with cytochrome c release from mitochondria and apoptosis. Appropriate expression of dynamin-related protein (Drp1) and the outer mitochondrial membrane GTPase Mitofusin 2 (MFN2) are critical for the mitochondrial fission and fusion processes (Arnoult, 2007). Phosphorylation and degradation of MFN2 are essential for stress-induced mitochondrial fragmentation and activation of apoptotic pathways. Mechanistically, JNK activation regulates MFN2 phosphorylation at Ser27, and HUWE1 is in turn recruited to interact with MFN2 and mediate its degradation, which facilitates stress-induced mitochondrial fragmentation and apoptotic cell death (Leboucher et al., 2012). HUWE1 was also demonstrated to play important roles in modulating the mitochondrial damage-induced mitophagy pathway by ubiquitinating the MFN2 protein. Mitophagy is essential for the removal of damaged mitochondria, and the proautophagic molecule autophagy/beclin-1 regulator-1 (AMBRA1) functions as a critical regulator of mitophagy by inducing proteasomal degradation of MFN2. HUWE1 was identified as an AMBRA1-interacting protein by mass spectrometry (MS) analysis. HUWE1 is translocated to mitochondria upon mitophagy stimulation and mediates the ubiquitination of MFN2 for its degradation. In addition, HUWE1 can mediate AMBRA1 phosphorylation and mitophagy initiation (Di Rita et al., 2018). Mechanistically, AMBRA1-induced mitophagy is regulated *via* the HUWE1-mediated Mcl-1 pathway. Mcl-1 phosphorylation recruits HUWE1 to interact with AMBRA1 on the mitochondrial membrane. In turn, HUWE1 regulates Mcl-1 ubiquitylation and degradation in response to AMBRA1-mediated mitophagy initiation (Strappazzon et al., 2020). Regarding the mTORC1-regulated autophagy process, mTORC1 phosphorylates WIPI2 at Ser395. HUWE1 was identified to interact with phosphorylated WIPI2 and mediate its degradation. Thus, HUWE1 inhibition resulted in increased autophagy (Wan et al., 2018). This study demonstrated that phosphorylation is essential for ubiquitination. In addition to playing a role in autophagy regulation, HUWE1 is also required for the degradation of unassembled proteins. HUWE1 was identified to mediate the degradation of unassembled Ubl4A protein and form the unassembled soluble protein degradation (USPD) system involved in the cytosolic protein quality control network (Xu et al., 2016). Overall, these findings indicate that HUWE1 regulates multiple signaling pathways, mitochondrial function, autophagy, cell metabolism, cell cycle progression and DNA damage responses to control apoptosis and cell proliferation and is thus involved in tumorigenesis modulation from initiation to progression to metastasis (Gong et al., 2020).

## HUWE1 promotes protein degradation during spermatogenesis and oocyte maturation

During spermatogenesis, histone proteins are degraded to permit chromatin condensation during spermatid elongation and maturation. E3^histones^ (HUWE1) was initially identified as an E3 ligase that mediates the ubiquitination of histone H2A (Liu et al., 2005). Moreover, the expression of E3^histones^ was found to be dynamically regulated over time within the testis during spermatogenesis, consistent with its function in histone ubiquitination and degradation involved in spermatid formation (Liu et al., 2007). During early embryonic development, siRNA-mediated knockdown of HUWE1 induced apoptotic cell death and inhibited normal embryonic development. Consistent with this finding, decreased HUWE1 staining in human embryos was found to be associated with poor embryo quality, suggesting that HUWE1 plays important roles in promoting embryonic development (Chen et al., 2016). Recently, an increasing number of mechanisms by which HUWE1 regulates germ cell development have been uncovered. HUWE1 deficiency in male germ cells was found to result in increased histone H2AX expression and an enhanced DNA damage response, which led to degeneration of spermatogonia and entry into meiosis (Bose et al., 2017; Fok et al., 2017). These studies collectively revealed that HUWE1 plays important roles in spermatogonial differentiation by orchestrating DNA damage responses. During oocyte maturation and preimplantation embryo development, oocyte-specific HUWE1 deletion was found to cause oocyte death and female infertility in mice, which were not rescued by p53 deletion, suggesting that another HUWE1 substrate might contribute to this pathway (Eisa et al., 2020).

## HUWE1 is associated with intellectual disability and regulates neural development

A large body of literature has shown that HUWE1 copy number variations are linked with intellectual disability (Giles and Grill, 2020), and HUWE1 is considered a dosage-sensitive gene associated with this phenotype (Froyen et al., 2012). Next-generation exome sequencing revealed HUWE1 as an X-linked intellectual disability (XLID) gene commonly identified in individuals with Juberg-Marsidi or Brooks syndrome (Friez et al., 2016). The authors identified the same mutation in the *HUWE1* gene in 2 XLID syndromes that were originally considered separate entities. In addition, more *HUWE1* missense mutations and splice site variants were reported in a large cohort of patients with intellectual disability harboring *HUWE1* variants, including in 14 females and 7 males (Moortgat et al., 2018). Recently, another novel splice site variant of the *HUWE1* gene was also identified in Say-Meyer syndrome by exome sequencing (Muthusamy et al., 2020). To date, mutations have been identified broadly across the HUWE1 protein sequence, and the C-terminal HECT domain and the N-terminal DUF908 domain are two mutation hotspots (Giles and Grill, 2020). Overall, HUWE1 has been recognized as the most prominent candidate gene linked to multiple intellectual disability syndromes, although defining the functional roles of HUWE1 in the intelligence genotype-phenotype correlation remains challenging. Continued human genetic and clinical studies, along with studies of the mechanisms by which HUWE1 regulats neuronal function and development, need to be further explored.

HUWE1 is a conserved regulator of neural progenitor proliferation and differentiation, cell migration and axon development in both vertebrate and invertebrate *in vivo* model systems (Giles and Grill, 2020). HUWE1 was found to regulate the transition of mouse hippocampal stem cells from a proliferating state to quiescence. In hippocampal stem cells, HUWE1 was found to interact with Achaete-scute family bHLH transcription factor 1 (Ascl1) and mediate its degradation, which inhibited the accumulation of cyclin D proteins and cell proliferation. This study demonstrated that HUWE1 is an important regulator of the return of proliferating stem cells to a transient quiescent state, which is essential for long-term maintenance of stem cells (Urbán et al., 2016). The sonic hedgehog (SHH) transcription factor Atoh1 is important for the regulation of cerebellar granule neuron progenitor proliferation and differentiation. Phosphorylation of Atoh1 at S328 and S339 was found to be essential for its stability, and HUWE1 specifically interacted with phosphorylated Atoh1 and mediated its degradation. SHH signaling thus inhibited Atoh1 phosphorylation and protected Atoh1 from HUWE1-mediated degradation, which prevented neuronal differentiation. This study indicated that HUWE1 is an important regulator controlling the balance between the proliferation and differentiation of neuronal progenitors (Forget et al., 2014). Previous studies by Zhao et al. demonstrated that the HUWE1 substrate N-Myc contributes significantly to neural differentiation and proliferation. Precise regulation of N-Myc oncoprotein stability is essential for nervous system development and neuronal differentiation. HUWE1 was reported to interact with N-Myc and mediate its K48-linked polyubiquitination for degradation. Genetic deletion of the *Huwe1* gene impaired neuronal differentiation, while silencing of the *N-Myc* gene in HUWE1-deleted cells restored cell cycle exit and differentiation (Zhao et al., 2008). Furthermore, HUWE1 was identified as a master regulator of the balance between proliferation and differentiation during brain development, and HUWE1 knockout was associated with glioblastoma (GBM). HUWE1 was found to negatively regulate the stability of N-Myc, and HUWE1 deficiency resulted in increased N-Myc protein accumulation in the brain, which induced DLL3 expression and Notch signaling activation. Thus, HUWE1 functions as a tumor suppressor to inhibit neuronal proliferation and promote neurogenesis (Zhao et al., 2009). In addition to directly modulating apoptosis-related protein activity, HUWE1 silencing reduced the apoptosis of cerebral cortical neurons in an ischemia-reperfusion model by modulating JNK and p38 activity (He et al., 2019). Overall, the function of HUWE1 in neural development is most likely to be promoting cell differentiation instead of proliferation, although how this function contributes to intellectual disability remains largely unknown.

## HUWE1 mediates inflammasome activation and inflammatory responses

The regulation of NF-κB activation by ubiquitination has been extensively studied, and different types of polyubiquitin chains have been demonstrated to be critical for the regulation of NF-κB signaling activity. K48- and K63-linked chains are the two most abundant and best studied ubiquitin chain types in different steps of the NF-κB signaling pathway. Ohtake et al. reported that HUWE1 was able to mediate K48-K63 branched heterogeneous ubiquitin chains to TRAF6 to amplify NF-κB signaling in response to stimulation (Ohtake et al., 2016). Miz1 is a negative regulator of TNFα-induced JNK activation and apoptosis. HUWE1 was identified as a Miz-associated protein and was found to catalyze its K48-linked polyubiquitination for degradation in response to TNFα stimulation. Inhibition of HUWE1 resulted in decreased JNK activation and apoptotic cell death triggered by TNFα treatment (Yang et al., 2010). In sickle cell disease (SCD), loss of endothelial peroxisome proliferator-activated receptor γ (PPARγ) was found to reduce HUWE1 expression, and HUWE1 was identified to mediate the ubiquitination of p65 for degradation. Thus, overexpression and activation of PPARγ might have high potential for treating SCD-associated pulmonary hypertension through HUWE1-mediated downregulation of NF-κb signaling (Jang et al., 2021). Our recent study revealed that HUWE1 is a master regulator of the activation of multiple inflammasomes, including the NLRP3, AIM2, and NLRC4 inflammasomes. HUWE1 directly interacts with the receptors of NLRP3, AIM2, and NLRC4 and mediates K27-linked polyubiquitination to promote inflammasome assembly, which positively contributes to host defense against multiple kinds of bacterial infections (Guo et al., 2020a). This was the first study to show that HUWE1 mediates K27-linked ubiquitination, which contributes to signaling activation. Moreover, interestingly, HUWE1 was identified as a novel substrate of caspase-1 through the Sensing EndoPeptidase Activity *via* Release and recapture using flAnking Tag Epitopes (SEPARATE) technique (Román-Meléndez et al., 2020). Together, these studies indicate the complex modulatory relationship between HUWE1 activity and inflammasome activation. Type I interferon signaling and interferon-stimulated genes (ISGs) form a backbone of the innate immune system. ISG proteins are a heterogenous group and need to interact with other cellular proteins to achieve full activity. In addition to mediating inflammasome activation and NF-κB signaling, HUWE1 was also identified by ISGs affinity proteomics to interact with ISG proteins, suggesting that HUWE1 might play essential roles in the ISG network upon infection and stress stimulation (Hubel et al., 2019). In immune thrombocytopenic purpura (ITP) patients, HUWE1 expression in CD4^+^ T cells in peripheral blood was increased. HUWE1 was identified to interact with E26 transformation-specific-1 (Ets-1) and facilitate its ubiquitination and degradation, which inhibited the differentiation of Treg cells and induced the immune imbalance observed in ITP (Li et al., 2021). HUWE1 is considered a potential candidate contributor to X chromosome- and sex-linked susceptibility to infectious diseases (Schurz et al., 2019). Overall, these studies indicate that HUWE1 might play both positive and negative roles in the activation of inflammatory signaling in a context- and substrate-dependent manner. The reported substrates targeted by HUWE1 are summarized in **Table 1**.

**Table 1:** Known Substrates of HUWE1.

Substrates	Ubiquitination type	Alternated gene name	Activity	References
**Cdc6**	Polyubiquitination	HUWE1	mediates Cdc6 degradation, promotes cell cycle arrest and apoptosis	Hall et al., 2007
**Mcl-1**	K48-linked polyubiquitination	Mule	promotes Mcl-1 degradation	Zhong et al., 2005
**H2AX**	K48-linked polyubiquitination	HUWE1	promotes H2AX degradation	Zhong et al., 2005
**PCNA**	Only interaction	HUWE1	contributes to H2AX mono-ubiquitination and phosophorylation	Choe et al., 2016
**hitone H1**	mono-ubiquitination	HUWE1	primes H1 ubiquitination for activating DNA damage response	Mandemaker et al., 2017
**Myst2**	ubiquitination	HUWE1	meidates degradatio of Myst2	Cho et al., 2020
**Pol β**	Polyubiquitination	Mule	promotes Pol βdegradation	Parsons et al., 2009
**Pol λ**	Polyubiquitination	Mule	promotes Pol λ degradation	Markkanen et al., 2012
**Chk1**	Polyubiquitination	HUWE1	mediates Chk1 degradation	Cassidy et al., 2020
**DDIT4**	Polyubiquitination	HUWE1	mediates DDIT4 degradation	Thompson et al., 2014
**Gadd45b**	Polyubiquitination	HUWE1	mediates Gadd45b degradation	He et al., 2015
**DNA-PKcs**	Neddylation	HUWE1	mediates DNA-PKcs neddylation to promote its phosphorylation and DNA repair	Guo et al., 2020b
**MYC**	K63-linked polyubiquitination	HectH9	promotes MYC activation	Adhikary et al., 2005
**c-Myc**	Polyubiquitination	HUWE1	promotes c-Myc degradation and suprresses prostate cancer cell	Qu et al., 2018
**p53**	K48-linked polyubiquitination	UREB1 (colorectal cancer)	promotes p53 degradation and functions as oncogene	Yoon et al., 2005
**p53**	polyubiquitination	ARF-BP1 (tumor cells)	function as tumor suppressor in p53-null cells; and oncogene in p53 wild-type cells	Chen et al., 2005
**c-Myc and Miz**	K48-linked polyubiquitination	HUWE1	promotes c-MYC and Miz degradation	Inoue et al., 2013
**TopBP1**	Polyubiquitination	HectH9	promotes TopBP1 degradation	Herold et al., 2008
**HDAC2**	polyubiquitination	Mule	promotes HDAC2 degradation	Zhang et al., 2011
**H1.3**	Polyubiquitination	HUWE1	promotes H1.3 degradation	Yang et al., 2017
**CTCF**	K48-linked polyubiquitination	ARF-BP1	promotes CTCF degradation	Qi et al., 2012
**Dvl**	K63-linked polyubiquitination	HUWE1	inhibits Dvl multimerization and the WNT signaling	De Groot et al., 2014
**MyoD**	Polyubiquitination	HUWE1	promotes MyoD degradation	Noy et al., 2012
**BRCA1**	polyubiquitination	HUWE1	promotes BRCA1 degradation	Wang et al., 2014
**TIAM1**	Polyubiquitination	HUWE1	promotes TIAM1 degradation	Page et al., 2007
**EGFR**	polyubiquitination	HUWE1	promotes EGFR degradation	Zhu et al., 2020
**HK2**	K63-linked polyubiquitination	HectH9	promotes HK2 activiation and tumor progression	Lee et al., 2019
**HIF-1α**	Polyubiquitination	HUWE1	promotes HIF-1α degradation	Zhang et al., 2020
**HAUSP**	K63-linked polyubiquitination	HectH9	promotes HAUSP activation	Wu et al., 2016
**MFN2**	Polyubiquitination	HUWE1	promotes MFN2 degradation	Leboucher et al., 2012
**WIPI2**	Polyubiquitination	HUWE1	promotes WIPI2 degradation	Wan et al., 2018
**Ubl4A**	Polyubiquitination	HUWE1	promotes unassembled Ubl4A degradation	Xu et al., 2016
**Histones (H1, H2A, H2B, H3, and H4)**	Polyubiquitination	E3^Histone^	promtotes H2A degradation	Liu et al., 2005
**Ascl1**	Polyubiquitination	HUWE1	promotes Ascl1 degradation	Urbán et al., 2016
**Atoh1**	Polyubiquitination	HUWE1	promotes Atoh1 degradation	Forget et al., 2014
**N-Myc**	Polyubiquitination	HUWE1	promotes N-Myc degradation	Zhao et al., 2008
**TRAF6**	K48-K63 branched ubiquitination	HUWE1	amplifies NF-κB signaling	Ohtake et al., 2016
**Miz1**	K48-linked polyubiquitination	HUWE1	reduces TNFα-induced JNK activation and apoptosis	Yang et al., 2010
**p65**	Polyubiquitination	HUWE1	promotes p65 degradation	Jang et al., 2021
**NLRP3**	K27-linked polyubiquitination	HUWE1	promotes NLRP3 activation	Guo et al., 2020a
**AIM2**	K27-linked polyubiquitination	HUWE1	promotes AIM2 activation	Guo et al., 2020a
**NLRC4**	K27-linked polyubiquitination	HUWE1	promotes NLRC4 activation	Guo et al., 2020a
**Ets-1**	Polyubiquitination	HUWE1	promotes Ets-1 degradation	Li et al., 2021
**TfR1**	Polyubiquitination	HUWE1	promotes TfR1 degradation	WU et al., 2022

## HUWE1 activity regulation and perspectives

**Figure 2:**
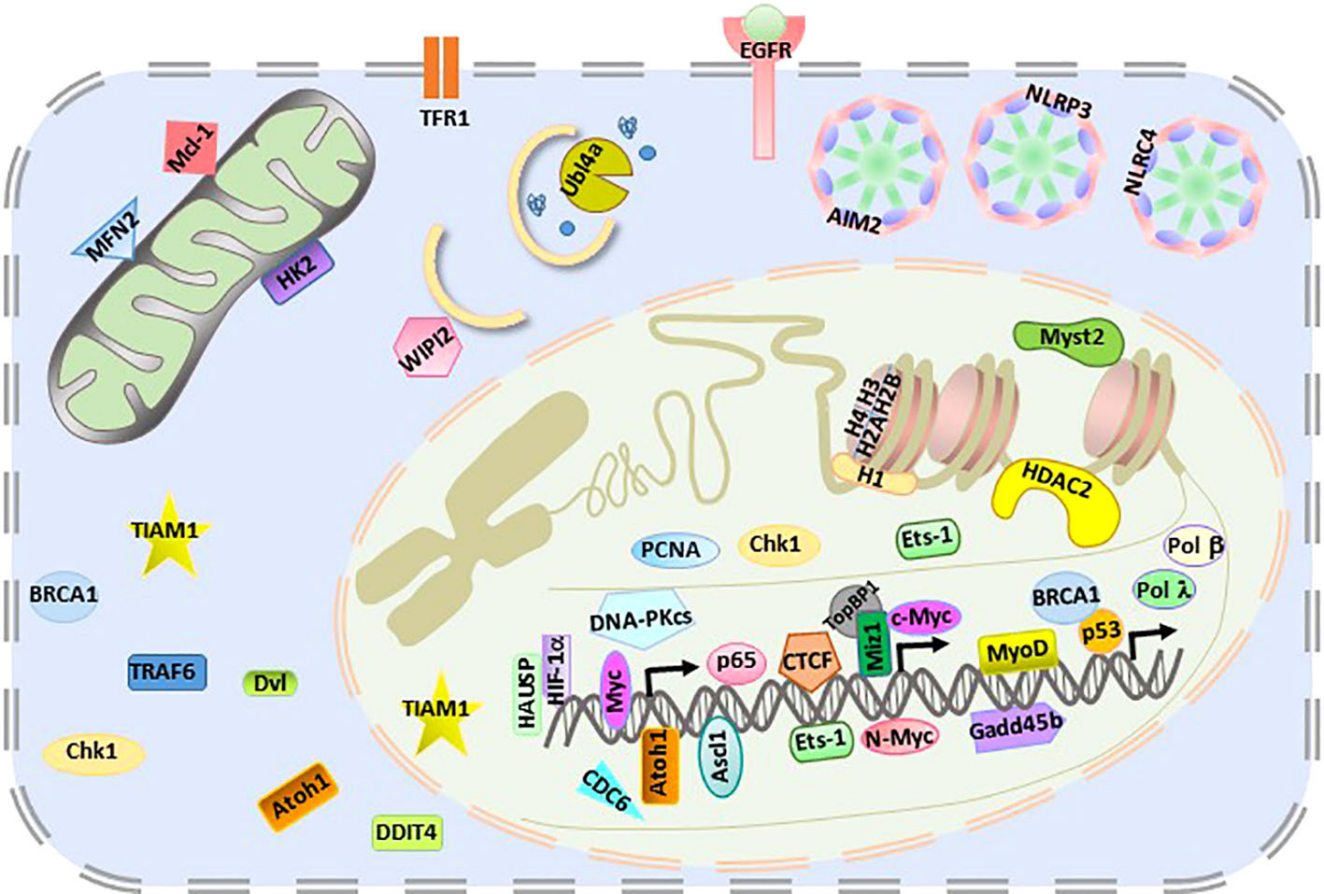
Intracellular localization of HUWE1 substrates.

The giant E3 ligase HUWE1 has been shown to extensively regulate more than 40 substrates, including membrane-bound receptors, cytosolic receptors, mitochondria-localized proteins, autophagy regulators, nuclear transcription factors, and chromatin epigenetic regulators (**Figure 2**). Furthermore, HUWE1 exhibits ligase activity in multiple cell types, including epithelial cells, tumor cells, neural cells, stem cells, germ cells, and immune cells. In a specific type of cell under different conditions, HUWE1 needs to target appropriate substrates to perform its function. How the precise regulation of HUWE1 is achieved remains unclear. In addition, the expanding atlas of HUWE1 substrates poses a major challenge to the potential application of HUWE1 in disease therapeutics.

HUWE1 activity might be regulated at multiple levels, for example, by transcriptional, posttranscriptional, and posttranslational modifications. Mcl-1 and PP5 function as antiapoptotic proteins and play critical roles in controlling cell proliferation and tumor development. MDM2 was also reported to mediate HUWE1 degradation and in turn regulate the abundance of Mcl1 and PP5 to contribute to tumor cell drug resistance (Kurokawa et al., 2013). The expression of MDM2 and HUWE1 in breast cancer and liposarcoma was demonstrated to have an inverse association, indicating the important role of the MDM2/HUWE1 axis in cell death regulation regardless of p53 status (Canfield et al., 2016). In addition, HUWE1 stability was found to be regulated by the E3 ligase CUL4B in response to DNA damage (Yi et al., 2015).

The HECT domain of HUWE1 is the primary domain controlling its ligase activity and substrate specificity, and an N-terminal helix element significantly stabilizes HECT domain activity (Pandya et al., 2010). Crystal structure analysis of the HUWE1 C-terminus revealed that dimerization inhibits HUWE1 activity, and that disruption of the dimer interface releases this inhibition. This study demonstrated that a conformational switch regulates HUWE1 activity (Sander et al., 2017). Recently, cryo-EM structures of *Nematocida* and human full-length HUWE1 demonstrated that HUWE1 harbors a snake-like and giant substrate-binding ring, which is highly dynamic, enabling engagement with a multitude of diverse substrates for catalysis *via* the flexible HECT domain (Hunkeler et al., 2021; Grabarczyk et al., 2021). The HUWE1 protein has become an attractive potential therapeutic target for treating cancer and other diseases, and structural analysis will significantly advance the development of activity inhibitors with substrate-selective recruitment. Bicyclic peptides and ubiquitin variants have been found to be able to efficiently and selectively inhibit HECT ligase activity, and HECT domains have been demonstrated to be potentially druggable (Mund et al., 2014; Zhang et al., 2016). In colorectal carcinogenesis, Peter et al. identified two specific HUWE1 inhibitors, BI8622 and BI8626, using high-throughput screening; these inhibitors were efficient in inhibiting MYC activation in colon cancer cells but not in normal colon cells. Furthermore, stabilization of MIZ induced by HUWE1 inhibition was found to result in suppression of MYC target gene expression (Peter et al., 2014). This study notably showed that the HUWE1 inhibitors BI8622 and BI8626 have prominent effects on the specific inhibition of HUWE1 activity and therapeutic potency. In addition, our study proved the inhibitory activity of BI8622 and BI8626 against HUWE1 in the inflammasome activation pathway and the potential therapeutic applications of BI8622 and BI8626 in treating inflammatory diseases (Guo et al., 2020a). Very recently, HUWE1 was demonstrated to target transferrin receptor 1 (TfR1) for degradation and inhibit ferroptosis in hepatocytes (Wu et al., 2022). Despite the significant advances achieved in identifying HUWE1 targets, our current understanding of HUWE1 activity regulation, particularly the temporal and spatial modulation of HUWE1 activation, is limited. Several important questions need to be examined in the future. The interplay and cross talk of the intrinsic functions of HUWE1 in immune cells and nonimmune cells in a particular disease model and the contribution of HUWE1-mediated immune responses to disease outcomes need to be carefully addressed. The essential differences and relationship among the substrates of HUWE1, together with the regulation of HUWE1 activity also deserve further exploration for potential therapeutic application.

## Author contributions

LQ, XX, and XQ conceived the review and wrote the paper. All authors contributed to the article and approved the submitted version.

## Funding

Studies in our lab are supported by the National Natural Science Foundation of China (82125021, 31970896, and 82072255).

## Conflict of interest

The authors declare that the research was conducted in the absence of any commercial or financial relationships that could be construed as a potential conflict of interest.

## Publisher’s note

All claims expressed in this article are solely those of the authors and do not necessarily represent those of their affiliated organizations, or those of the publisher, the editors and the reviewers. Any product that may be evaluated in this article, or claim that may be made by its manufacturer, is not guaranteed or endorsed by the publisher.
